# Estimation of Congestion in Free Disposal Hull Models Using Data Envelopment Analysis

**DOI:** 10.1155/2014/427673

**Published:** 2014-10-15

**Authors:** M. Abbasi, G. R. Jahanshahloo, M. Rostamy-Malkhlifeh, F. Hosseinzadeh Lotfi

**Affiliations:** Department of Mathematics, Islamic Azad University, Science and Research Branch, Tehran 1477893855, Iran

## Abstract

This paper deals with evaluating congestion in free disposal hull (FDH) models. There are several approaches in data envelopment analysis (DEA) literatures which discuss the theory and application of congestion. However, almost all of these approaches considered convex DEA technologies. So, in the case of nonconvex technologies, including FDH technology, this field is almost nil. This paper makes an attempt to fill in this void. To do so, this study provides a pairwise comparisons-based algorithm to evaluate congestion in FDH model. This algorithm identifies the sources of congestion and estimates its amounts. It is also capable of detecting the losses amounts of output due to congestion. The validity of the proposed model is demonstrated using some numerical and empirical examples.

## 1. Introduction

Evaluation of decision making units (DMUs) is an important task especially from a managerial point of view. DEA is a nonparametric and mathematical programming based approach for evaluating the performance of a set of homogeneous DMUs using multiple inputs to produce multiple outputs. In performance analysis, in particular in DEA, the concept of congestion plays a seminal role in theory and application. Congestion is a special phenomenon in the production process which is defined in economics where outputs are reduced due to excessive amount of inputs or an increase in one or more outputs results in a reduction in one or more inputs. For an actual example of congestion in a coal mine where a large crowd of the miners are working in a narrow underground, the amount of minerals excavated will be reduced [1].

Heretofore, various approaches have been presented in DEA for the treatment of congestion. The concept of congestion was first introduced in the literatures by Färe and Grosskopf [2] in the context of DEA. Subsequently an operationally implementable form was given by Färe et al. [3] and Cooper et al. [4–6]. Afterwards, Tone and Sahoo [7] developed a new slack-based approach to evaluate the scale elasticity in the presence of congestion with a unified framework. Wei and Yan [8] used DEA output additive models and proposed a necessary and sufficient condition for existence of congestion. Jahanshahloo and Khodabakhshi [9, 10] provided an approach of input congestion based on the relaxed combinations of inputs. Later on, Khodabakhshi [11] provided a one-model approach of input congestion based on input relaxation model. Also Khodabakhshi [12] proposed a method to detect the input congestion in the stochastic DEA. To see more references about this approach, the readers are referred to [13, 14]. Jahanshahloo et al. [15] and Khodabakhshi et al. [16] proposed some methods for computing the congestion in DEA models with production trade-offs and weight restrictions. Sueyoshi and Sekitani [17] proposed a modified approach which is able to measure congestion under the occurrence of multiple solution. There exist some papers which reviewed congestion papers, as that of Khodabakhshi et al. [18].

All of the above-mentioned investigations deal with congestion in convex technologies. In convex models, the targets resulting from efficiency assessment correspond to the points on the continuous efficiency frontiers. This means that DMUs might be compared with unreal DMUs which sometimes is meaningless in real life, for example, when we want to evaluate the efficiency of various car engines.

FDH models were first formulated by Deprins et al. [19]. The PPS of FDH model is made by deterministic (or observed activities) and free disposability postulates. So the PPS of FDH model is nonconvex. One appealing characteristic of FDH model due to nonconvexity nature of FDH efficiency frontier is that, in FDH model, targets correspond to observed units which is more compatible with real life because, in some circumstances, the observed unit is more comfortable when compared with a real unit rather than with a virtual one. As can be seen from the foregoing, there are several methods for evaluating congestion in convex DEA models, but for FDH models, although there are a few papers which are concerned with the field of estimation returns to scale (RTS), see, for example, [20–24], methods to estimate congestion can be hardly found. Therefore a new scheme is required to deal with congestion in FDH models.

In this paper, we first present definitions of output efficiency for DMUs under a series of DEA output additive models. Then, using these definitions, we develop a necessary and sufficient condition for existence of congestion in FDH model. Afterwards, we provide a polynomial time algorithm based on pairwise comparisons which evaluates congestion for DMUs using certain differences of inputs and outputs. This algorithm simply identifies the sources of congestion and estimates its amounts for congested DMUs.

The rest of the paper unfolds as follows. In the next section, FDH model and some of its properties and definitions will be presented to facilitate later discussions. In Section 3, we present a method with many computational advantages for evaluating congestion in FDH model. The validity of the proposed model is demonstrated using three numerical examples in Section 4. Finally, Section 5 gives the conclusion of this paper.

## 2. Preliminaries

In this section we first briefly describe some characteristic property of FDH model. Consider *n* DMUs where each DMU_*j*_  (*j* = 1,…, *n*) utilizes *m* inputs *x*_*ij*_  (*i* = 1,…, *m*) to produce *s* outputs *y*_*rj*_  (*r* = 1,…, *s*). Let *x*_*j*_ = (*x*_1*j*_,…,*x*_*mj*_)^*T*^ and *y*_*j*_ = (*y*_1*j*_,…,*y*_*sj*_)^*T*^. We will also assume that *x*_*j*_ ≥ 0, *x*_*j*_ ≠ 0 and *y*_*j*_ ≥ 0, *y*_*j*_ ≠ 0. The production possibility set *T* is represented as

(1) $$\begin{array}{c} T=\left\{\left(x,y\right)\in R_{+}^{m+s}\;|\;y\;\text{can}\;\text{be}\;\text{produced}\;\text{from}\;x\right\}. \end{array}$$

Deprins et al. [19] have deduced the following production possibility set. This set is denoted by *T*_FDH_, regarding the assumptions of deterministic and free disposability of the production technology:

(2) TFDH={(x,y):∑j=1nλjxj≤x,∑j=1nλjyj≥y,  ∑j=1nλj=1,λj∈{0,1},j=1,…,n}.

The additive FDH model to evaluate the efficiency of a special DMU_*p*_  (*p* ∈ {1,…, *n*}) under the *T*_FDH_ is as follows:

(3) Max⁡ ∑i=1msi−+∑r=1ssr+,s.t.  ∑j=1nλjxij+si−=xip i=1,…,m,∑j=1nλjyrj−sr+=yrp r=1,…,s,∑j=1nλj=1, λj∈{0,1},  j=1,…,n,si+≥0 i=1,…,m,sr+≥0 r=1,…,s.

**Definition 1 (FDH efficiency).**

Consider model (3). If the optimal objective value is zero, then DMU_*p*_ is said to be FDH efficient.

It is worth noting that different to CCR and BCC models, the FDH model does not operate with the convexity assumption. Therefore, this model has a discrete nature which causes the efficient target point for an inefficient DMU simply to be assigned as a point among only actually observed DMUs. Thus, the efficiency analysis is done relative to the other given DMUs instead of a hypothetical efficiency frontier. This has the advantage that the achievement goal for an inefficient DMU given by its efficient target point will be more credible than in cases of CCR and BCC models.

**Definition 2 (FDH output efficiency).**

Consider the following model. If *Z*_FDH_ = 0, then DMU_*p*_ is said to be FDH output efficient:

(4) ZFDH=Max⁡ ∑r=1ssr+ s.t.  ∑j=1nλjxij≤xip i=1,…,m    ∑j=1nλjyrj−sr+=yrp r=1,…,s    ∑j=1nλj=1, λj∈{0,1},  j=1,…,n,    sr+≥0 r=1,…,s.

**Definition 3 (congestion).**

Evidence of congestion is present in the performance of any DMU, when a decrease in one or more inputs is associated with increases that are maximally possible in one or more outputs without worsening other inputs or outputs. Conversely, congestion is said to occur when some of the outputs that are maximally possible are reduced by increasing one or more inputs without improving any other inputs or outputs [25].

A very restrictive form of the above definition yields the definition of strong congestion as follows.

**Definition 4 (strong congestion).**

If a proportionate reduction in all inputs of a DMU warrants an increase in all maximally possible outputs, then strong congestion occurs [7].

**Definition 5 (technical efficiency).**

Efficiency is achieved by DMU_0_ if and only if it is not possible to improve some of its inputs or outputs without worsening some of its other inputs or outputs [25].

**Definition 6 (technical inefficiency).**

Technical inefficiency is said to be present in the performance of DMU_0_ when the evidence shows that it is possible to improve some input or output without worsening some other inputs or outputs [25].

## 3. Congestion in FDH Model

**Figure 1:**
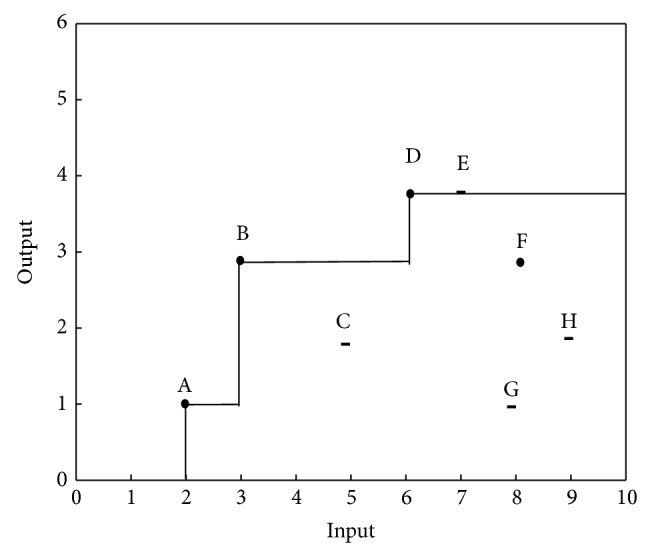
*T*_FDH_.

In *T*_FDH_, the efficiency surface is a staircase based on those given DMUs that are not dominated by other given DMUs.  Figure 1 describes an illustrative example of *T*_FDH_ which is made by eight DMUs denoted by A, B,…, H with one input and one output.

It should be noted that evaluating congestion in customary models for convex PPS has been studied on *T*_NEW_, which is a PPS without input disposability postulate. Let us denote *T*_NEW_ corresponding to *T*_FDH_ as *T*_NFDH_, which can be defined as follows:

(5) TNFDH={(x,y) ∣ x=∑j=1nλjxj,y≤∑j=1nλjyj,  ∑j=1nλj=1,λj∈{0,1},j=1,…,n}.

**Figure 2:**
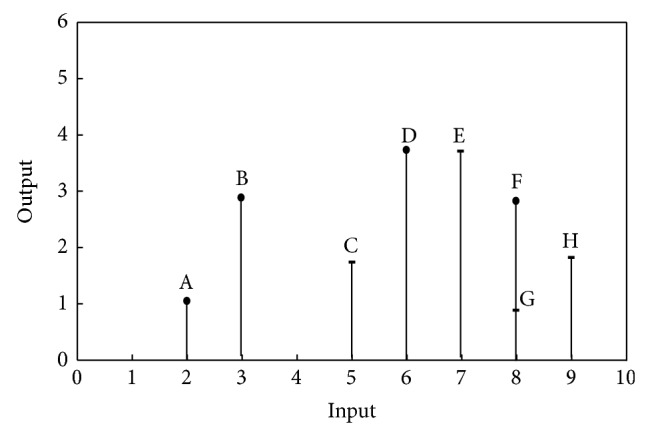
*T*_NFDH_.

Figure 2 exhibits *T*_NFDH_ for the example in Figure 1. As seen from Figure 2, *T*_NFDH_ has a discrete nature and so the study of congestion on it is complicated. So to overcome this difficulty we introduce a new set as follows:

(6) FDH−1={(x,y) ∣ x≤∑j=1nλjxj,y≤∑j=1nλjyj,  ∑j=1nλj=1,λj∈{0,1},j=1,…,n}.

**Figure 3:**
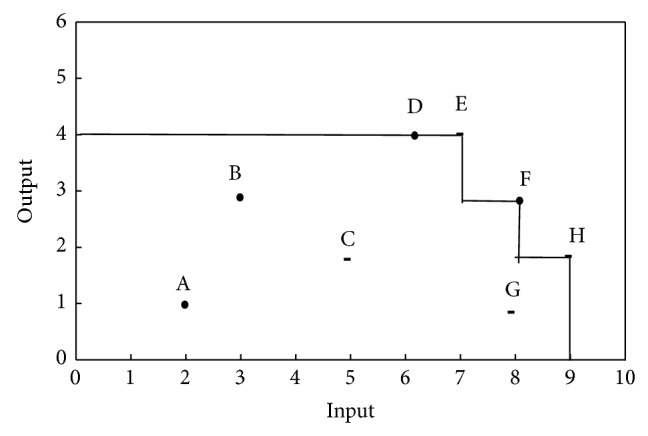
FDH^−1^.

Apparently, the set of FDH^−1^ is gained by reversing the sign of input inequalities in *T*_FDH_. FDH^−1^ set corresponding to the example in Figure 1 is illustrated in Figure 3.

We use the following model to deal with the congestion phenomenon in FDH model:

(7) ZFDH−1=Max⁡ ∑r=1ssr+ s.t.  ∑j=1nλjxij≥xip i=1,…,m    ∑j=1nλjyrj−sr+=yrp r=1,…,s    ∑j=1nλj=1, λj∈{0,1},  j=1,…,n    sr+≥0 r=1,…,s.

We call the above model “FDH^−1^ output additive model.”

To see what is involved, we note that the input (like the output) constraints take the form ∑_*j*=1_^*n*^ *λ*_*j*_ *x*_*ij*_ ≥ *x*_*ip*_. Hence, in this adaptation of additive models, the objective is to maximize the outputs without reducing any of the inputs.

**Definition 7 (*FDH* ^−1^ output efficiency).**

Consider the model (7). If *Z*_FDH^−1^_ = 0, then DMU_*p*_ is said to be FDH^−1^ output efficient.

**Lemma 8 .**

*DM* *U*_*p*_ is *FDH* ^−1^ output efficient if and only if the following system has no solution:

(8) $$\begin{array}{c} \overset{n}{\underset{j=1}{\sum }}\lambda_{j}x_{j}\geq x_{p},\\ \overset{n}{\underset{j=1}{\sum }}\lambda_{j}y_{j}\geq y_{p},\;\;\overset{n}{\underset{j=1}{\sum }}\lambda_{j}y_{j}\neq y_{p},\\ \overset{n}{\underset{j=1}{\sum }}\lambda_{j}=1,\;\lambda_{j}\in \left\{0,1\right\},\;\;j=1,\ldots ,n. \end{array}$$

**Proof**

It is clear using Definition 7.

**Definition 9 (congestion in FDH model).**

Let DMU_*p*_ = (*x*_*p*_, *y*_*p*_) be FDH^−1^ output efficient; if there exists DMU_*k*_ = (*x*_*k*_, *y*_*k*_), such that *x*_*k*_ ≤ *x*_*p*_,  *x*_*k*_ ≠ *x*_*p*_ and *y*_*k*_ ≥ *y*_*p*_,  *y*_*k*_ ≠ *y*_*p*_, then DMU_*p*_ has evidence of congestion.

Based upon Definition 9, units F, G, and H in Figure 3 have evidence of congestion and unit C is technically inefficient.

**Definition 10 (strong congestion in FDH model).**

Let DMU_*p*_ = (*x*_*p*_, *y*_*p*_) be congested in FDH model; if there exists DMU_*k*_ = (*x*_*k*_, *y*_*k*_), such that *x*_*k*_ < *x*_*p*_ and *y*_*k*_ > *y*_*p*_, then DMU_*p*_ has evidence of strong congestion.

**Lemma 11 .**

Let *DMU*_*p*_ be *FDH* ^−1^ output efficient; then *DMU*_*p*_ has evidence of congestion if and only if the following system has a solution:

(9) $$\begin{array}{c} \overset{n}{\underset{j=1}{\sum }}\lambda_{j}x_{j}\leq x_{p},\\ \overset{n}{\underset{j=1}{\sum }}\lambda_{j}y_{j}\geq y_{p},\;\;\overset{n}{\underset{j=1}{\sum }}\lambda_{j}y_{j}\neq y_{p},\\ \overset{n}{\underset{j=1}{\sum }}\lambda_{j}=1,\;\lambda_{j}\in \left\{0,1\right\},\;\;j=1,\ldots ,n. \end{array}$$

**Proof**

Let DMU_*p*_ has evidence of congestion, so from Definition 9, there exists DMU_*k*_ = (*x*_*k*_, *y*_*k*_), such that *x*_*k*_ ≤ *x*_*p*_,  *x*_*k*_ ≠ *x*_*p*_ and *y*_*k*_ ≥ *y*_*p*_, *y*_*k*_ ≠ *y*_*p*_. Thus *λ*_*k*_ = 1 and *λ*_*j*_ = 0  (*j* = 1,…, *n*, *j* ≠ *k*) is a solution of (9).

Conversely, suppose that $\tilde{\lambda }=(0,\ldots ,0,1,0,\ldots ,0)$, whose qth component is one, is a solution of (9). So, we have *x*_*q*_ ≤ *x*_*p*_ and *y*_*q*_ ≥ *y*_*p*_,  *y*_*q*_ ≠ *y*_*p*_. Also, certainly *x*_*q*_ ≠ *x*_*p*_, since, according to the assumption of lemma, DMU_*p*_ is FDH^−1^ output efficient, so *x*_*q*_ = *x*_*p*_ contradicts Lemma 8. Hence, by Definition 9, DMU_*p*_ has evidence of congestion.

**Lemma 12 .**

*DM* *U*_*p*_ is not FDH output efficient if and only if the following linear system has a solution:

(10) $$\begin{array}{c} \overset{n}{\underset{j=1}{\sum }}\lambda_{j}x_{j}\leq x_{p},\\ \overset{n}{\underset{j=1}{\sum }}\lambda_{j}y_{j}\geq y_{p},\;\;\overset{n}{\underset{j=1}{\sum }}\lambda_{j}y_{j}\neq y_{p},\\ \overset{n}{\underset{j=1}{\sum }}\lambda_{j}=1,\;\lambda_{j}\in \left\{0,1\right\},\;\;j=1,\ldots ,n. \end{array}$$

**Proof**

Using definition of FDH output efficiency, the proof is completed.

We now present the main result of the proposed method.

**Theorem 13 .**

Let *DMU*_*p*_ be *FDH* ^−1^ output efficient; then *DMU*_*p*_ has evidence of congestion if and only if *DMU*_*p*_ is not FDH output efficient.

**Proof**

Using Lemmas 11 and 12, the proof is completed.

Now, using Theorem 13, we can provide the following procedure to evaluate congestion in FDH model.

1. Solve model (7) corresponding to (*x*_*p*_, *y*_*p*_); let (*λ* ^*^, *s* ^+∗^) be the optimal solution of it. Let ${\hat{y}}_{p}=y_{p}+{s^{+}}^{*}$. It is evident that $\left(x_{p},{\hat{y}}_{p}\right)$ is FDH^−1^ output efficient.
2. Solve model (4) for $\left(x_{p},{\hat{y}}_{p}\right)$.
3. If *Z*_FDH_ > 0, then DMU_*p*_ is congested.

**Remark 14 .**

Models (4) and (7) are mixed-integer programming, but we can simply show that it does not need any mathematical programming problem to solve. Indeed, an enumeration algorithm based on pairwise comparisons, similar to Tulken's enumeration algorithm for the case of radial FDH model [26], can be used.

Now, based upon foregoing procedure and Remark 14, we propose the following algorithm. The proposed algorithm includes two parts. In Part (a), we recognize the existence of congesting in performance of DMU_*p*_ and in Part (b), if DMU_*p*_ is recognized to be congested in Part (a), the amount of congestion for each input as well as the reduction amount of each output due to congestion will be estimated.

*Proposed Algorithm*

*Part (a)*

**Step 1 .**

Calculate the optimal value of model (7) by the following equation:

(11) $$\begin{array}{c} Z_{\text{FD}{\text{H}}^{-1}}=\overset{s}{\underset{r=1}{\sum }}\left(y_{rq}-y_{rp}\right)=\underset{j\in D_{p}}{\max}\;\overset{s}{\underset{r=1}{\sum }}\left(y_{rj}-y_{rp}\right), \end{array}$$

where

(12) $$\begin{array}{c} D_{p}=\left\{j\in \left\{1,\ldots ,n\right\}\;|\;x_{j}\geq x_{p}\;\;\text{and}\;\;y_{j}\geq y_{p}\right\}. \end{array}$$

**Step 2 .**

Let ${\hat{y}}_{p}=y_{p}+s^{+*}$, where *s* ^+∗^ = *y*_*q*_ − *y*_*p*_. Obtain the optimal value of model (4) by

(13) $$\begin{array}{c} Z_{\text{FDH}}=\underset{j\in {\hat{D}}_{p}}{\max}\;\overset{s}{\underset{r=1}{\sum }}\left(y_{rj}-{\hat{y}}_{rp}\right), \end{array}$$

where

(14) $$\begin{array}{c} {\hat{D}}_{p}=\left\{j\in \left\{1,\ldots ,n\right\}\;|\;x_{j}\leq x_{p}\;\;\text{and}\;\;y_{j}\geq {\hat{y}}_{p}\right\}. \end{array}$$

**Step 3 .**

If *Z*_FDH_ > 0, then DMU_*p*_ is congested, so go to Part (b); furthermore, if there exist $j\in {\hat{D}}_{p}$ such that *x*_*j*_ < *x*_*p*_ and $y_{j}>{\hat{y}}_{p}$, then, based on Definition 4, DMU_*p*_ is strongly congested. If *Z*_FDH_ = 0, then DMU_*p*_ is not congested and stop.

*Part (b)*

**Step 4 .**

Define *K*_*p*_ as follows:

(15) $$\begin{array}{c} K_{p}=\left\{j\in {\hat{D}}_{p}\;|\;Z_{\text{FDH}}=\overset{s}{\underset{r=1}{\sum }}\left(y_{rj}-{\hat{y}}_{rp}\right)\right\}. \end{array}$$

Then calculate

(16) $$\begin{array}{c} \alpha^{*}=\underset{j\in k_{p}}{\min}\;\overset{m}{\underset{i=1}{\sum }}\left(x_{ip}-x_{ij}\right). \end{array}$$

**Step 5 .**

Define *T*_*p*_ as follows:

(17) $$\begin{array}{c} T_{p}=\left\{j\in K_{p}\;|\;\alpha^{*}=\overset{m}{\underset{i=1}{\sum }}\left(x_{ip}-x_{ij}\right)\right\}. \end{array}$$

For *j* ∈ *T*_*p*_ define *s*_*i*_^c^^*^ as the amount of congestion in *i*th input of DMU_*p*_ and ${\hat{s}}_{r}^{+*}$ as reduction amount of *r*th output due to congestion as follows:

(18) $$\begin{array}{c} s_{i}^{c*}=x_{ip}-x_{ij},\;i=1,\ldots ,m,\\ {\hat{s}}_{r}^{+*}=y_{rj}-{\hat{y}}_{rp},\;r=1,\ldots ,s. \end{array}$$

*α* ^*^ = ∑_*i*=1_^*m*^ *s*_*i*_^*c*∗^ is the total amount of congestion in all inputs of DMU_*p*_.

**Corollary 15 .**

If ${\hat{D}}_{p}=\emptyset$, congestion has no appearance at *DMU*_*p*_.

**Proof**

In this case, it is obvious that system (10) has no solution. So, by Lemma 12, $\left(x_{p},{\hat{y}}_{p}\right)$ is FDH output efficient and regarding Theorem 13 congestion has no appearance at $\left(x_{p},{\hat{y}}_{p}\right)$. Since congestion is a frontier concept, then DMU_*p*_ has no congestion

**Remark 16 .**

If Card({*j* ∈ *D*_*p*_∣*Z*_FDH^−1^_ = ∑_*r*=1_^*s*^(*y*_*rj*_ − *y*_*rp*_)}) > 1, then the projection of DMU_*p*_, $\left(x_{p},{\hat{y}}_{p}\right)$, is not determined uniquely.

**Remark 17 .**

Card(*T*_*p*_) ≥ 1. If Card(*T*_*p*_) = 1, then the amount of congestion can be uniquely determined; otherwise there are alternatives for the amount of congestion for DMU_*p*_.

Using the above algorithm, we can evaluate the congestion for each DMU in FDH model without any mathematical programming problems and with only some certain pairwise differences of inputs and outputs with regard to (11), (16), and (18). Therefore, the following theorem is obviously true.

**Theorem 18 .**

The proposal algorithm is a polynomial time algorithm.

## 4. Numerical Examples

In this section, we apply our proposed procedure to measure the congestion effect on two numerical examples and an empirical example.

**Example 1 .**

We consider the illustrative example provided in Section 3 which includes eight DMUs, A, B, C, D, E, F, G, and H, with one output and one input each, as shown in Figure 1. The data set of DMUs as well as the results of Part (a) of the proposed algorithm is given in Tables 1 and 2 displays results of Part (b). As shown in Table 1, there is no congestion in DMUs A, B, C, D, and E and congestion has appeared in DMUs F, G, and H. The input congestion amount and reduction amount of output due to congestion for congested units, resulting from the proposed algorithm, have been provided in the two last columns of Table 2, respectively. Obviously, in the case of one input and one output, each congested unit has evidence of strong congestion.

**Example 2 .**

We consider an example adopted from Tone and Sahoo ([7], page 756) which has been listed in Table 3 of our study. This example consists of four DMUs, A, B, C, and D, using two inputs and producing two outputs. The results of Part (a) of the proposed algorithm are given in Table 3. From Table 3 we can see that there is no congestion in DMUs A and B. As shown in Table 4, for unit C, the congestion amount of inputs is (*s*_1_^*c*∗^, *s*_2_^*c*∗^) = (0,1) and $\left({\hat{s}}_{1}^{+*},{\hat{s}}_{2}^{+*})=(0,1\right)$ is output losses of C due to congestion. For unit D, the congestion amount of inputs is (*s*_1_^*c*∗^, *s*_2_^*c*∗^) = (1,1) and $\left({\hat{s}}_{1}^{+*},{\hat{s}}_{2}^{+*})=(1,1\right)$ is output losses of D due to congestion. Therefore considering Definition 10, unit D is strongly congested.

**Example 3 .**

Table 5 presents the input and output data of the Chinese textile industry during 1981–1997 assembled by Cooper et al. [5]. Each year has been treated as a DMU with two inputs and one output: labor (*X*_1_) measured in units of 1000 persons, capital (*X*_2_) measured in units of one million Ren Min Be (Chinese monetary unit), and output (*Y*) measured in units of one million Ren Min Be too. Note that the capital and output values have been adjusted to a 1991 base period to eliminate the impact of price variations. The congestion amount regarding FDH technology using the proposed method as well as the congestion amounts using Cooper et al.'s method [6] in BCC technology which are shown by (*s*_1_^*c*∗^, *s*_2_^*c*∗^) is provided in Table 6. As can be seen from Table 6, congestion appeared in performance of DMUs 8, 9, 10, and 15 in *T*_FDH_. Besides DMUs 2, 8, 9, 10, 12, 13, and 15 have been recognized to be congested in *T*_BCC_. Comparison of these two computational results shows that the DMUs which are congested in *T*_FDH_ are among the ones that are congested in *T*_BCC_. That is, the set of congested DMUs in *T*_FDH_ is a subset of set of congested DMUs in *T*_BCC_. It seems reasonable because *T*_FDH_⊆*T*_BCC_.

## 5. Conclusion

In this paper we proposed a method based on pairwise comparison to evaluate congestion in FDH model. The results of the study have been proved with some lemmas and theorems. Our proposed method is able to identify congestion in performance of DMUs and it can determine the amount of excessive inputs for congested DMUs based on the calculation of certain pairwise differences of inputs and outputs. It also is capable of detecting the losses amounts of output due to congestion. One of the advantages of this method is that in all stages there is no need to solve any mathematical programming problems and so a polynomial time algorithm to identify congestion in FDH model is provided. Hence, it is superior from a computational point of view. The numerical examples demonstrated the compatibility of the proposed approach and so can be developed in performance analysis and large practical projects.

Because of having low complexity of computation, this method can be developed for imprecise data for further research.

## Figures and Tables

**Table 1:** Data set and results of Part (a) of the proposed algorithm in Example 1.

DMUs	*I*	*O*	*D*_*p*_	*Z*_FDH^−1^_	*s* ^+^^*^	${\hat{y}}_{p}$	${\hat{D}}_{p}$	*Z*_FDH_	Status
A	2	1	{A, B, C, D, E, F}	3	3	4	{A}	0	No congestion
B	3	3	{B, D, E, F}	1	1	4	{B}	0	No congestion
C	5	2	{C, E, F, H}	2	2	4	{}		No congestion
D	6	4	{D, E}	0	0	4	{D}	0	No congestion
E	7	4	{E}	0	0	4	{D}	0	No congestion
F	8	3	{F}	0	0	3	{B, D, E}	1	Congestion
G	8	1	{F, G, H}	2	2	3	{B, D, E, F}	1	Congestion
H	9	2	{H}	0	0	2	{B, C, D, E, F}	2	Congestion

**Table 2:** Results of Part (b) of the proposed algorithm in Example 1.

DMUs	*k*_*p*_	*α* ^*^	*T*_*p*_	*s* ^c^^*^	${{\hat{s}\;}^{+}}^{*}$
F	{E, D}	1	{E}	1	1
G	{E, D}	1	{E}	1	1
H	{E, D}	2	{E}	2	2

**Table 3:** Data set and results of Part (a) of the proposed algorithm in Example 2.

DMUs	(*I*_1_, *I*_2_)	(*O*_1_, *O*_2_)	*D*_*p*_	*Z*_FDH^−1^_	(*s*_1_^+^^*^, *s*_2_^+^^*^)	${\hat{y}}_{p}$	${\hat{D}}_{p}$	*Z*_FDH_	Status
A	(1, 1)	(1, 1)	{A, B, C, D}	2	(1, 1)	(2, 2)	{}		No congestion
B	(2, 2)	(2, 2)	{B}	0	(0, 0)	(2, 2)	{B}	0	No congestion
C	(2, 3)	(2, 1)	{C}	0	(0, 0)	(2, 1)	{B, C}	1	Congestion
D	(3, 3)	(1, 1)	{D}	0	(0, 0)	(1, 1)	{A, B, C, D}	2	Congestion

**Table 4:** Result of Part (b) of the proposed algorithm in Example 2.

DMUs	*K*_*p*_	*α* ^*^	*T*_*p*_	(*s*_1_^c^^*^, *s*_2_^c^^*^)	$({{\hat{s}\;}_{1}^{+}}^{*}$, ${{\hat{s}\;}_{2}^{+}}^{*})$
C	{B}	1	{B}	(0, 1)	(0, 1)
D	{B}	2	{B}	(1, 1)	(1, 1)

**Table 5:** Data set in Example 3.

DMU = year	Labor (*X*_1_)	Capital (*X*_2_)	Output (*Y*)
DMU_1_ = 1981	389.00	19.86	856.02
DMU_2_ = 1982	412.30	21.16	866.85
DMU_3_ = 1983	423.50	17.08	956.04
DMU_4_ = 1984	417.30	18.10	1082.94
DMU_5_ = 1985	570	12.61	1273.20
DMU_6_ = 1986	600.50	13.45	1230.72
DMU_7_ = 1987	641.10	15.91	1410.66
DMU_8_ = 1988	715.30	23.72	1728.16
DMU_9_ = 1989	736.00	25.97	2109.57
DMU_10_ = 1990	745.00	18.24	2291.08
DMU_11_ = 1991	756.00	14.40	2533.27
DMU_12_ = 1992	743.00	17.50	2899.16
DMU_13_ = 1993	684.00	25.08	3520.74
DMU_14_ = 1994	691.00	25.45	4949.93
DMU_15_ = 1995	673.00	29.35	4604.00
DMU_16_ = 1996	634.00	23.05	4722.29
DMU_17_ = 1997	596.00	25.02	4760.28

**Table 6:** Comparison results of congestion treatment in BCC and FDH technologies in Example 3.

DMU = year	(*s*_1_^*c*∗^, *s*_2_^*c*∗^) in FDH	(*s*_1_^*c*∗^, *s*_2_^*c*∗^) in BCC	Results of the proposed approach in *T*_FDH_	Results of Cooper et al.'s approach in *T*_BCC_ [6]
DMU_1_ = 1981	(0, 0)	(0, 0)	No congestion	No congestion
DMU_2_ = 1982	(0, 0)	(0, 0.72)	Congestion	No congestion
DMU_3_ = 1983	(0, 0)	(0, 0)	No congestion	No congestion
DMU_4_ = 1984	(0, 0)	(0, 0)	No congestion	No congestion
DMU_5_ = 1985	(0, 0)	(0, 0)	No congestion	No congestion
DMU_6_ = 1986	(0, 0)	(0, 0)	No congestion	No congestion
DMU_7_ = 1987	(0, 0)	(0, 0)	No congestion	No congestion
DMU_8_ = 1988	(81.30, 0.67)	(65.39, 0)	Congestion	Congestion
DMU_9_ = 1989	(45.00, 0.52)	(45.00, 0)	Congestion	Congestion
DMU_10_ = 1990	(2.00, 0.74)	(43.16, 0)	Congestion	Congestion
DMU_11_ = 1991	(0, 0)	(0, 0)	No congestion	No congestion
DMU_12_ = 1992	(0, 0)	(30.72, 0)	No congestion	Congestion
DMU_13_ = 1993	(0, 0)	(1.79, 0)	No congestion	Congestion
DMU_14_ = 1994	(0, 0)	(0, 0)	No congestion	No congestion
DMU_15_ = 1995	(7.70, 4.33)	(0, 3.98)	Congestion	Congestion
DMU_16_ = 1996	(0, 0)	(0, 0)	No congestion	No congestion
DMU_17_ = 1997	(0, 0)	(0, 0)	No congestion	No congestion
